# Natural Aminoacyl tRNA Synthetase Fragment Enhances Cardiac Function after Myocardial Infarction

**DOI:** 10.1371/journal.pone.0109325

**Published:** 2014-10-08

**Authors:** Margaret E. McCormick, Mauricio Rojas, Tyler Moser-Katz, Ellie Tzima, John S. Reader

**Affiliations:** 1 Department of Cell Biology and Physiology, University of North Carolina at Chapel Hill, Chapel Hill, North Carolina, United States of America; 2 UNC McAllister Heart Institute, University of North Carolina at Chapel Hill, Chapel Hill, North Carolina, United States of America; Tokai University, Japan

## Abstract

A naturally-occurring fragment of tyrosyl-tRNA synthetase (TyrRS) has been shown in higher eukaryotes to ‘moonlight’ as a pro-angiogenic cytokine in addition to its primary role in protein translation. Pro-angiogenic cytokines have previously been proposed to be promising therapeutic mechanisms for the treatment of myocardial infarction. Here, we show that systemic delivery of the natural fragment of TyRS, mini-TyrRS, improves heart function in mice after myocardial infarction. This improvement is associated with reduced formation of scar tissue, increased angiogenesis of cardiac capillaries, recruitment of c-kit^pos^ cells and proliferation of myocardial fibroblasts. This work demonstrates that mini-TyrRS has beneficial effects on cardiac repair and regeneration and offers support for the notion that elucidation of the ever expanding repertoire of noncanonical functions of aminoacyl tRNA synthetases offers unique opportunities for development of novel therapeutics.

## Introduction

The primary functional role of the aminoacyl-tRNA synthetases (aaRSs) is to ligate specific amino acids to their canonical tRNAs in the first step of protein synthesis. In the last decade, there has been the growing realization that these enzymes have a wide range of additional cell signaling functions outside of their canonical ‘housekeeping’ roles in higher eukaryotes [1]. Non-canonical functions of aaRSs include regulation of angiogenesis, inflammation and tumorigenesis, while genetic mutations in human aaRSs are now thought of as causative factors for the pathology of several diseases [2]. The first example of a ‘moonlighting’ aaRS was human cytosolic tyrosyl-tRNA synthetase (TyrRS) [3], which is secreted from endothelial cells (ECs) as a natural fragment – mini-TyrRS [4]. Once outside the confines of the cell, mini-TyrRS can activate angiogenic signaling pathways in ECs *in vitro* and stimulate angiogenesis *in vivo* [5], as well as function as a chemo-attractant to white blood cells [3]. The ever-expanding roles of aaRSs and their fragments (now branded Physiocrines) in human physiology and pathology hold the promise of their use as therapeutic reagents and pharmacological targets.

Heart disease is a leading cause of death in developed countries; acute occlusion of a major coronary artery leads to cardiac ischemia due to reduction or elimination of blood flow, loss of cardiac tissue, and its subsequent replacement with non-functional scar tissue [6]. Although the area that experiences complete loss of blood supply suffers irreversible damage, the myocardium surrounding the infarct zone is thought to be capable of recovery if angiogenesis and restoration of blood flow occur [7], [8]. In this vein, efforts have focused on the timely revascularization of the ischemic myocardium by delivery of angiogenic and chemotactic molecules, including vascular endothelial growth factor (VEGF), basic fibroblast growth factor (bFGF, FGF2), stromal cell derived factor-1 (SDF-1), hepatocyte growth factor (HGF), to name a few [9]–[11]. For example, cardiac-specific over-expression of FGF2 results in enhanced recovery of cardiac contractile function and reduced infarct size after ischemia-reperfusion injury [12], while increased expression of nerve growth factor (NGF) promotes angiogenesis and survival of cardiomyocytes after MI and leads to increased levels of c-kit^pos^ progenitor cells in hearts [13]. Similar c-kit^pos^ cell homing to infarcted hearts was noted in response to stromal cell-derived factor-1α [8], [14]. Additionally, several studies have utilized stem cells, both embryonic stem cells (ESCs) and induced pluripotent stem cells (iPSCs), as well as epicardium derived cells, to regenerate the heart [15]–[22].

It is universally accepted that ECs play a crucial role in regulating the survival and function of neighboring cardiomyocytes by promoting angiogenesis and vascular regeneration, but also by regulating cardiomyocyte contractility and growth via paracrine signaling [23]. Signaling downstream of mini-TyrRS can trigger EC migration, proliferation and angiogenesis [4]. On the basis of these properties of mini-TyrRS and its previously reported chemotactic activity, we investigated the potential for mini-TyrRS to promote murine cardiac recovery and function in response to coronary occlusion. We found that mice treated with mini-TyrRS after MI do indeed have improved recovery of heart function. These changes in hearts in response to mini-TyrRS are associated with a decrease in cardiac fibrosis, an increase in angiogenesis of cardiac capillaries, proliferation of cardiac fibroblasts and increased levels of c-kit^pos^ progenitor cells in hearts.

## Materials and Methods

### Animals

Adult (12 weeks), male C57Bl/6 mice were purchased from Jackson Laboratories (Bar Harbor, ME, USA).

### Ethics statement

Animals were used in accordance with the guideline of the National Institute of Health and for the care and use of laboratory animals (procedures were approved by the Institutional Animal Care and Use Committees of the University of North Carolina at Chapel Hill).

### Echocardiography

Conscious echocardiography was performed as previously described using a Vevo 2100 ultrasound biomicroscopy system [24]. All LV dimension data are presented as the average of at least 3 independent waveforms.

### Left anterior descending coronary artery ligation (LAD) and mini-TyrRS treatment

Mouse surgeries were performed as previously described [25]. Briefly, mice were anesthetized with isoflurane and a thoracotomy was performed for ventilation. The chest was then opened, the peri-cardial sac opened and the LAD artery was permanently occluded with a suture. For sham injury, an identical procedure was followed. Body temperature was monitored with a rectal probe. Endotoxin-free, recombinant mini-TyrRS (250 ng/g), prepared as previously described [26], or bacteriostatic saline was injected IP daily on days 0–6 after surgery.

### Histological analysis and Immunofluorescence

Hearts were perfused and fixed with fresh 4% paraformaldehyde for 24 hours. The hearts were then paraffin embedded and sectioned into 5 µm sections and stained with hematoxylin and eosin or Masson’s trichrome to assess overall morphology [27]. For determination of scar area, ImageJ software was used to trace the fibrotic area (blue) from an image taken through the middle of the LV and expressed as fibrotic area relative to total area. For determination of capillary density, heart sections were stained with TRITC-conjugated lectin (*Triticum vulgaris*) and examined by fluorescence microscopy as previously described [24], [27]. Specifically, for each heart section (10 images/mouse), the number of lectin-positive vessels surrounding round cardiomyocytes was counted. These values were then averaged to yield the average vessel density per cardiomyocyte. For immunohistochemistry, antibodies used were: CD45 (rat), BD Pharmingen; CC3 (rabbit), Cell Signaling; PCNA, Abcam; c-kit (H300) (rabbit); vimentin (goat), Santa Cruz.

### Quantitation and statistical analysis

Probability values were obtained by performing a 2-tailed Student *t*-test; statistical significance was defined as *P<*0.05.

## Results

### Systemic mini-TyrRS delivery improves cardiac function after infarction

**Figure 1:**
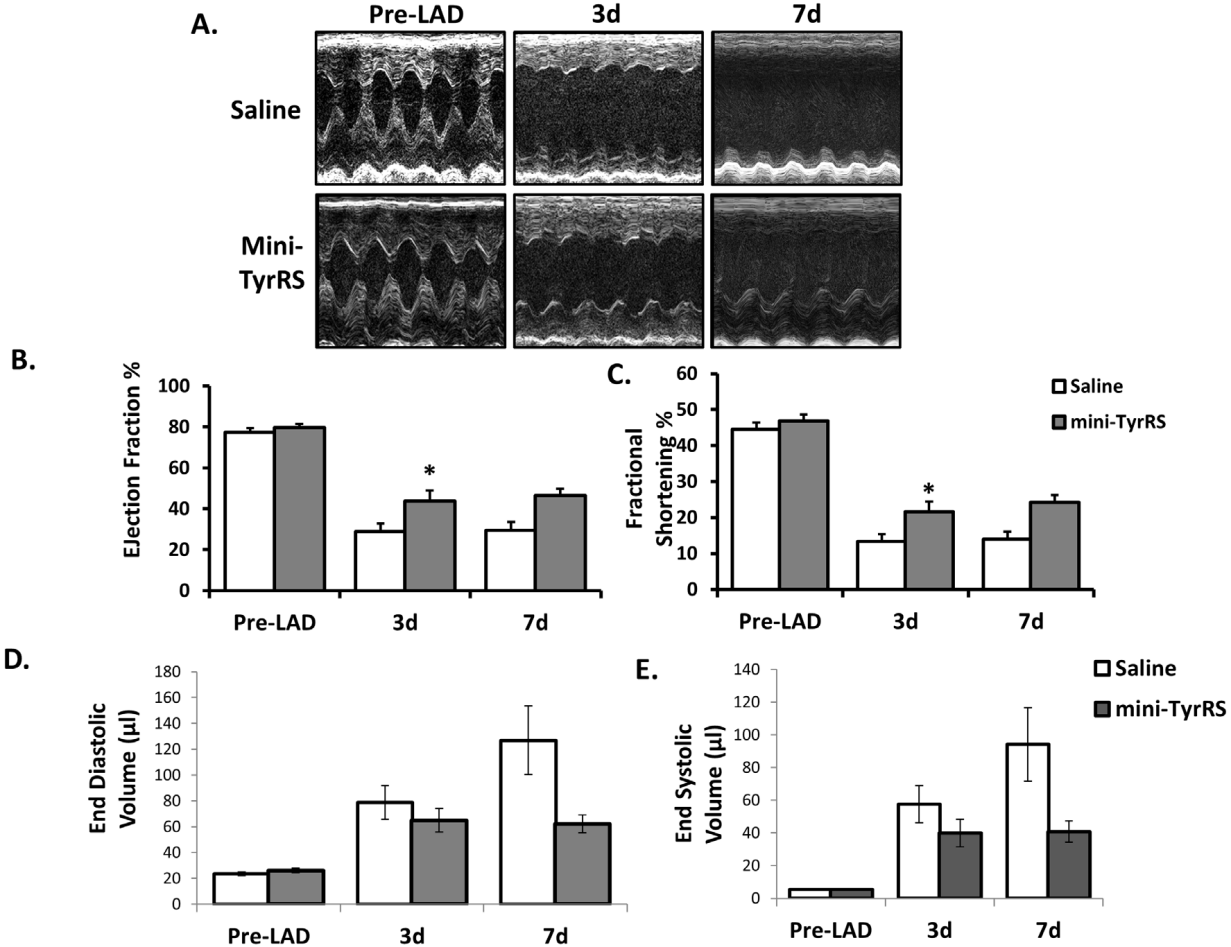
Mini-TyrRS treatment after coronary ligation improves myocardial function *in vivo*. A. Representative images of M-Mode echocardiography for heart function of saline- and mini-TyrRS treated mice at 3d and 7d post MI. B–C. Left ventricle fractional shortening and ejection fraction after coronary artery ligation with or without mini-TyrRS treatment. Echocardiography results shown are mean ± SEM (n = 7–12 mice per group; *P*<0.03). Measurements were collected at the level of the papillary muscle in M-mode. D–E. End Diastolic volume and end systolic volume after coronary artery ligation with or without mini-TyrRS treatment. Results shown are mean ± SEM (n = 7–12 mice per group). Although results did not reach statistical significance (*P*<0.09), there is a trend towards significance for decreased end diastolic and end systolic volumes after mini-TyrRS treatment compared to saline controls.

To test the hypothesis that mini-TyrRS could improve heart function after MI, we performed left anterior descending (LAD) coronary artery ligations in adult (12–14 weeks) male mice. Ligation of the LAD results in blockade of blood flow to a portion of the left ventricle (LV), thus creating a zone of ischemia. Following surgery, mice were given intraperitoneal (IP) injections of either sterile saline control or 250 ng/g mini-TyrRS for 3 days. Mice were subjected to conscious echocardiography to assess cardiac function either pre-LAD or 3 days (3d) and 7d after LAD (Table I). Although both treatment groups had a significant decrease in heart function in response to MI, mini-TyrRS-treated mice had attenuated LV dysfunction compared to the saline-treated group, as indicated by both fractional shortening (FS) and ejection fraction (EF) (Figure 1B, C; Table I). At 3d after infarction, LVs of saline treated mice had a mean FS of 13.4±1.92% (n = 8); whereas mini-TyrRS treatment resulted in a mean FS of 21.6±2.8% (n = 9). As a second measure of cardiac function, echocardiographic measurements revealed that the mean fraction of blood ejected from the LV in saline-treated mice was 28.8±3.9%, compared with a mean of 43.8±5% for the mini-TyrRS-treated group. At 7d after infarction we similarly observed an increase in cardiac function of mini-TyrRS-treated mice. FS was 14.0±2.08 (n = 9) for the saline-treated group and 24.2±1.99 (n = 7) for the mini-TyrRS-treated group (p = 0.077), and EF was 29.5±4.14 (n = 9) for the saline-treated group and 46.7±3.35 (n = 7) for the mini-TyrRS-treated group (p = 0.086) (Figure 1). Consistent with these results, end diastolic and end systolic volumes at 7d were lower in the mini-TyrRS-treated group compared to the saline-treated group (126.9 vs 62.1 µl and 94.2 vs 40.8 µl, respectively), although these values did not reach statistical significance due to the high variability amongst animals (p = 0.076 and p = 0.089, respectively) (Figure 1D). Importantly, we found that mini-TyrRS administration in the absence of infarction did not lead to an increase in cardiac function (Table I). Collectively, these results indicate that mini-TyrRS treatment leads to a significant improvement of murine heart function after myocardial infarction.

**Table 1:** Echocardiographic data from saline- and mini-TyrRS-treated hearts pre and post-LAD.

	Saline			Mini-TyrRS		
	Pre-LAD (n = 11)	3d (n = 8)	7d (n = 9)	Pre-LAD (n = 11)	3d (n = 9)	7d (n = 7)
**HR (BPM)**	653.4±21.8	644±23.6	685±15.5	688±11.3	674.5±15.1	657.7±6.29
**IVS; d (mm)**	1.01±0.03	0.74±0.06	0.72±0.08	1.00±0.01	0.85±0.09	0.67±0.03
**IVS; s (mm)**	1.56±0.04	0.90±0.11	0.99±0.12	1.58±0.03	1.14±0.09	0.92±0.06
**LVPW; d (mm)**	0.96±0.03	0.77±0.0	0.71±0.09	0.96±0.02	0.88±0.04	0.89±0.04
**LVPW; s (mm)**	1.36±0.04	0.93±0.07	0.82±0.10	1.47±0.05	1.11±0.06*	1.10±0.05*
**EDV (µl)**	23.5±1.5	78.8±13.1	126.0±26.7	26.5±1.7	64.8±9.0	62.1±6.89
**ESV (µl)**	5.4±0.7	57.6±11.4	94.2±22.4	5.5±0.7	39.9±8.4	40.8±6.4
**EF%**	77.3±2.1	28.8±3.9	29.5±4.1	79.6±1.7	43.8±5.0*	46.7±3.4*
**FS%**	44.5±1.9	13.4±1.9	14.0±2.1	46.8±1.7	21.6±2.8*	24.2±2.0*

HR, heart rate; IVS, interventricular septum; LVPW, left ventricular posterior wall; ESD, end-diastolic volume; ESV, end-systolic volume; EF, ejection fraction; FS, fractional shortening.

*P<0.05 vs. saline.

### Mini-TyrRS treated hearts have reduced levels of scar formation after infarction

**Figure 2:**
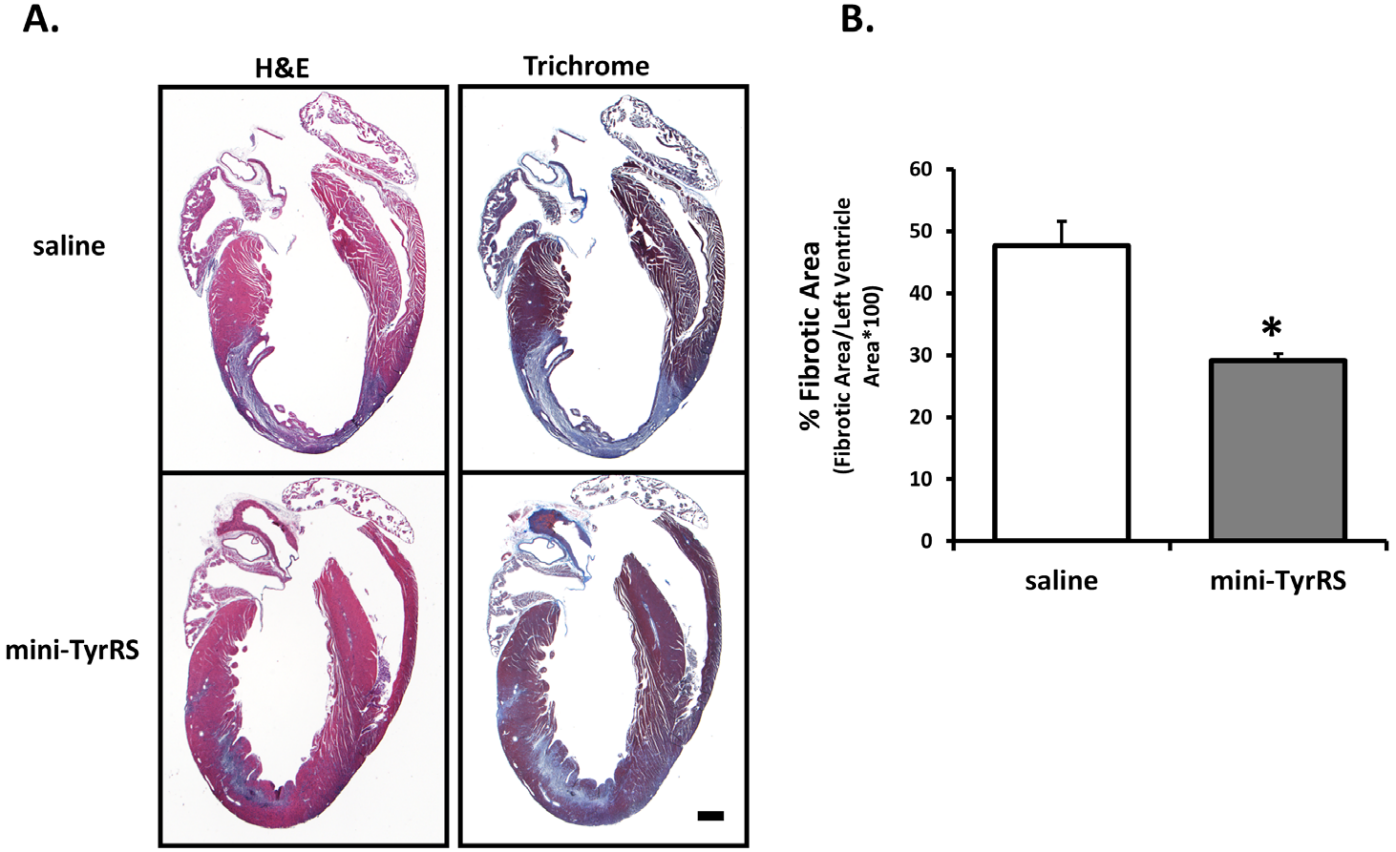
Mini-TyrRS reduces levels of scar formation after infarction. A. Representative H&E and Masson’s Trichrome stained sections 3d after coronary artery ligation and saline or mini-TyrRS treatment. B. Quantitation of scar tissue of hearts after coronary artery ligation; results are represented as fibrotic area/left ventricle area, mean ± SEM (n = 3 mice/group; 1 section taken from the middle of the LV was quantified/mouse, *P*<0.03). Bar is 50 um.

Myocardial infarction leads to hypoxic conditions in the heart and subsequent death of cardiomyocytes; to maintain the structural integrity of the heart after this significant tissue loss, cardiac fibroblasts migrate into the damaged infarct area and increase deposition of extracellular matrix components, including collagen, thus leading to a build up of non-contractile scar tissue in these recovering hearts [28]. Histological analysis of heart morphology and scar tissue formation by hematoxylin and eosin and Masson’s trichrome staining revealed a marked reduction in the size of the scar tissue area and LV wall that appeared to be thicker after mini-TyrRS treatment (Figure 2). The fibrotic area in mini-TyrRS-treated hearts was ∼61% relative to saline-treated controls. These results show that mini-TyrRS treatment of murine hearts after myocardial infarction is associated with a reduction in the levels of scar tissue formation and an accompanying thicker functional LV wall.

### Mini-TyrRS treatment after myocardial infarction results in enhanced angiogenesis

**Figure 3:**
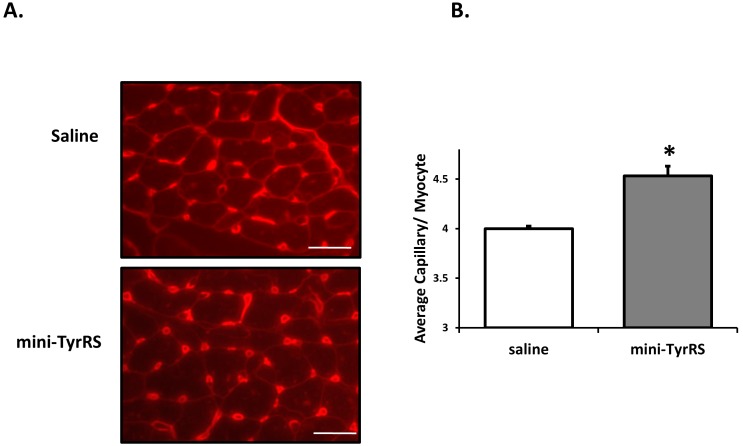
Mini-TyrRS treatment enhances vascularization after infarction. A. Representative TRITC-lectin stained sections 3d after coronary artery ligation and saline or mini-TyrRS treatment. B. Quantitation of capillaries per cardiomyocyte; values shown are mean ± SEM (n = 3 mice/group; 9–11 images were quantified/mouse; *P<0.04). Bar is 20 um.

Previous work with ECs showed that mini-TyrRS promotes angiogenesis by activating angiogenic signaling pathways within ECs [4], [5]. As vascularization of the heart is critical for cardiomyocyte survival, we hypothesized that mini-TyrRS induces neoangiogenesis. To test this, we harvested hearts and stained with TRITC-lectin to visualize capillaries within the ischemic border region. Quantification of the capillaries revealed a statistically significant increase in microvasculature in mini-TyrRS-treated hearts compared to the saline controls (Figure 3). These results suggest that the cardioprotective effects of mini-TyrRS may, in part, be attributed to increased cardiac neovascularization, and therefore perfusion, of the tissue surrounding the infarct.

### Mini-TyrRS reduces myocardial apoptosis after infarction

**Figure 4:**
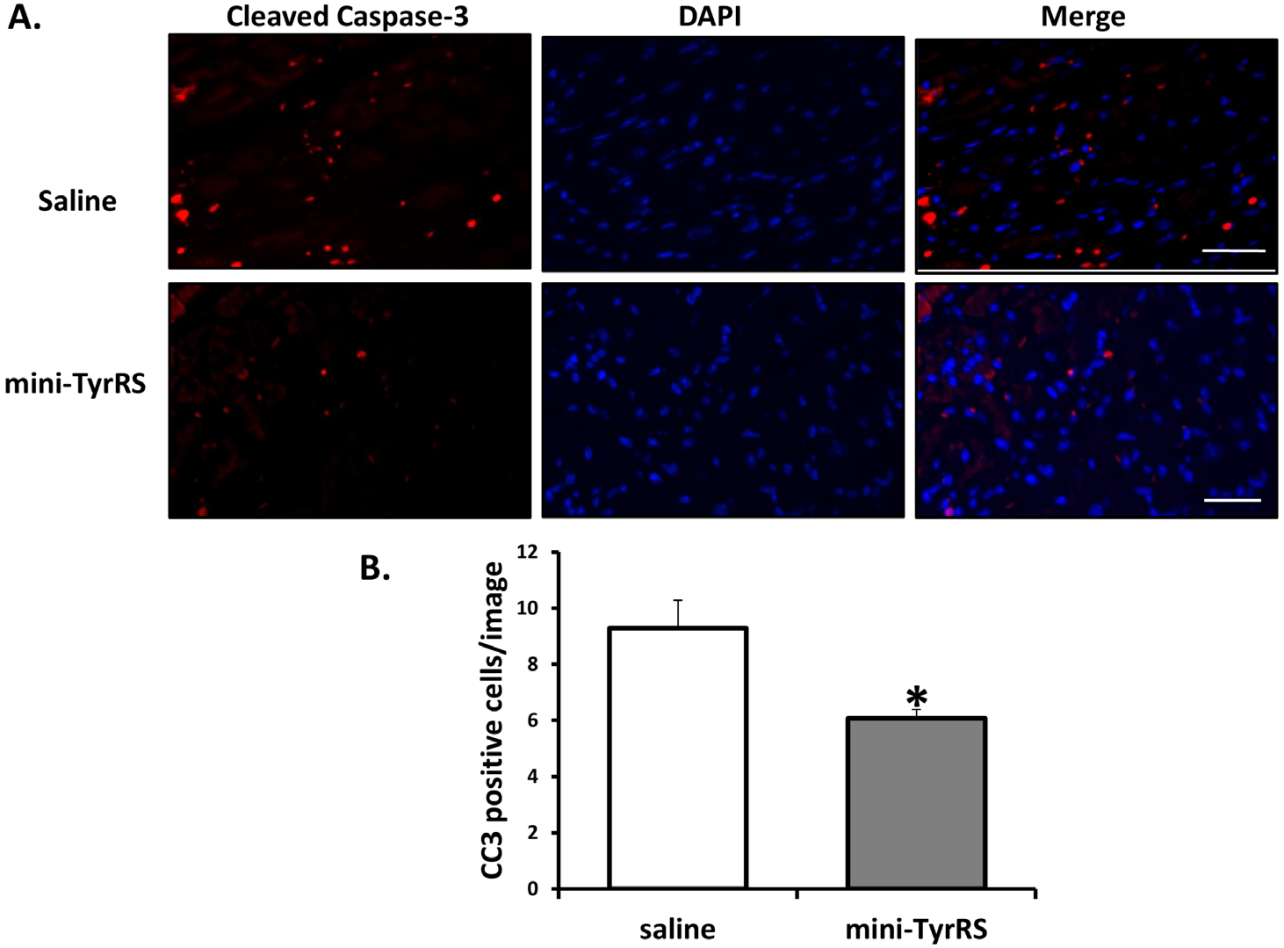
Mini-TyrRS promotes cell survival after coronary artery ligation. A. Representative cleaved caspase-3 (CC3) stained sections 7d after coronary artery ligation and saline or mini-TyrRS treatment. B. Quantitation of CC3 positive cells/image; values shown are mean ± SEM (n = 3 mice/group; 10 images were quantified/mouse; *P<0.05). Bar is 100 um.

To investigate further the mechanism by which mini-TyrRS improves cardiac function, we examined the degree of apoptosis in the infarct zone and the neighboring ischemic zone by immunostaining with anti-cleaved caspase 3 antibody (CC3). Quantitation revealed a significant reduction in the number of CC3-positive cells in the ischemic zone of mini-TyrRS-treated hearts, compared to saline-treated hearts (Figure 4). There was no detectable CC3 staining in areas remote from the ischemia for either treatment group (data not shown). Thus, mini-TyrRS protects the neighboring ischemic zone from apoptosis in response to infarction.

### Mini-TyrRS treatment promotes cardiac fibroblast proliferation after infarction

**Figure 5:**
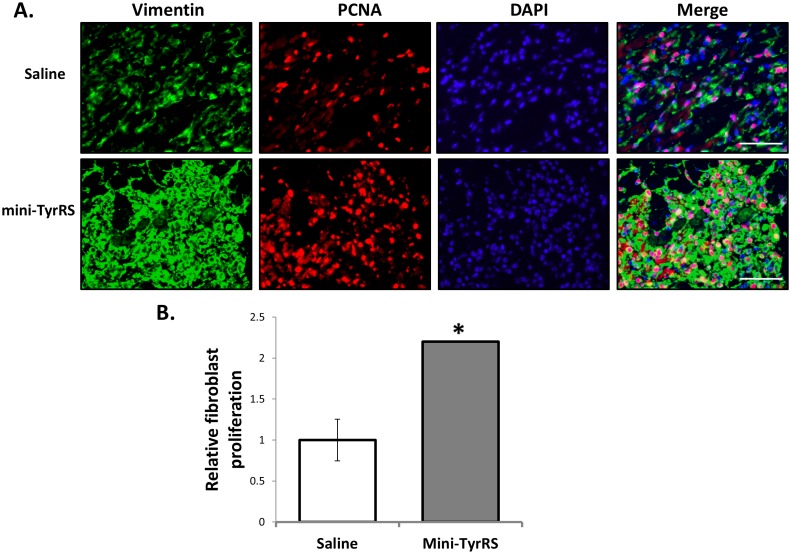
Mini-TyrRS treatment promotes cardiac fibroblast proliferation after infarction. A. Representative sections of hearts 3d after coronary artery ligation and saline or mini-TyrRS treatment. Sections were immunostained for vimentin, PCNA and DAPI. B. Quantitation of vimentin- and PCNA- double positive cells in saline and mini-TyrRS treated hears; values represent fold change relative to saline group; values shown are mean ± SEM (n = 3 mice/group; 10 images were quantified/mouse; *P<0.05). Please note the increased number of vimentin-positive cells infiltrating hearts of mini-TyrRS-treated animals. Bar is 100 um.

In response to cardiomyocyte necrosis in the infracted heart, cell migration and proliferation is associated with the healing phase of cardiac remodeling [29]. During this time, cardiac fibroblasts display high proliferative activity and migration through the matrix network [29]. We assayed cell proliferation at the border zone by nuclear proliferating cell nuclear antigen (PCNA) staining. Proliferation in the peri-infarct area was increased in mini-TyrRS-treated hearts compared to saline-treated hearts (Figure 5). As significant fibroblast proliferation has been documented in the infracted heart, we immunostained sections with an anti-vimentin antibody and quantitated the number of PCNA- and vimentin-positive cells; as seen in Figure 5, mini-TyrRS treatment resulted in significant increase in proliferating fibroblasts, compared to saline treatment.

### Effects of mini-TyrRS treatment on c-kit^pos^ cells in the heart after infarction

**Figure 6:**
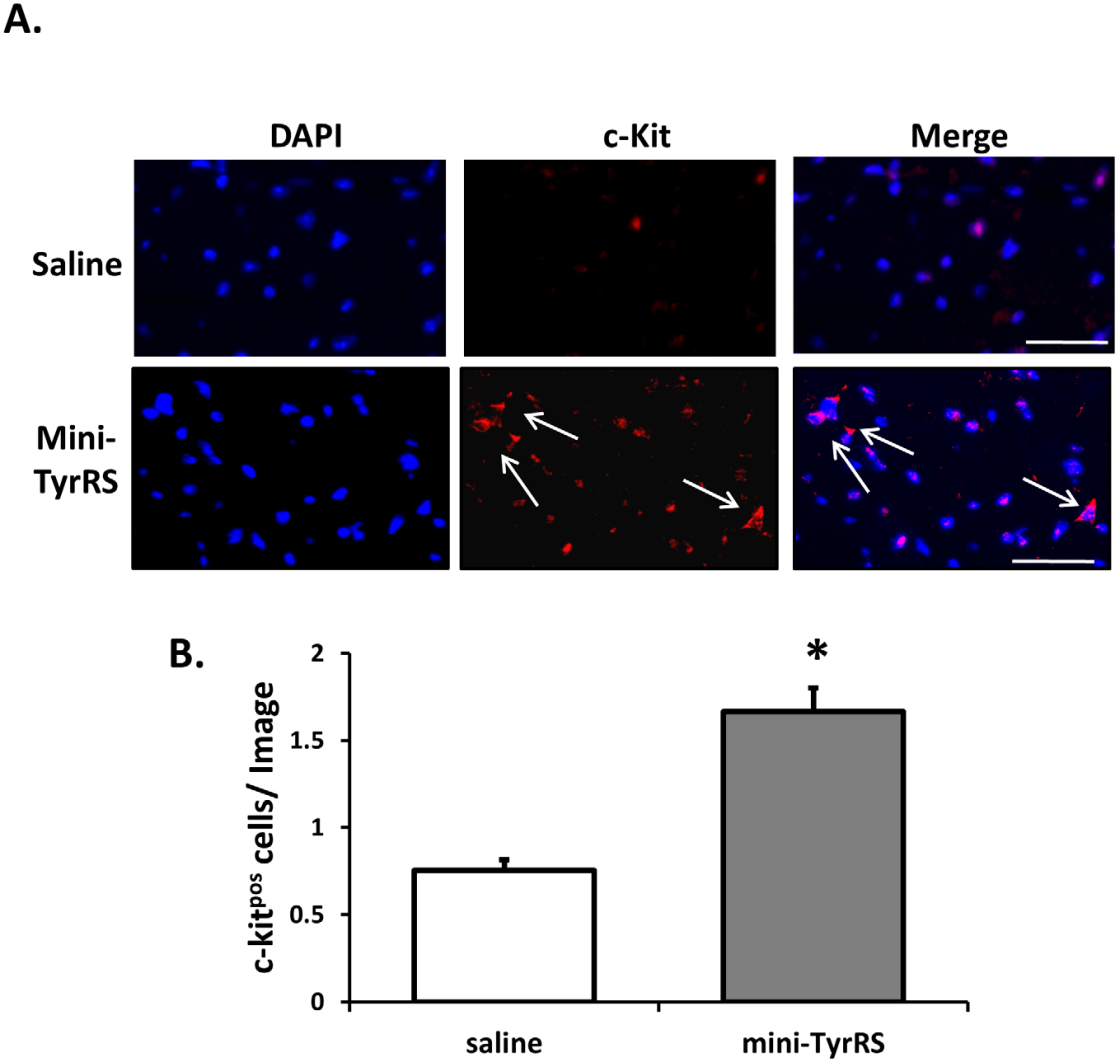
Increased c-Kit^pos^ cells in mini-TyrRS-treated hearts after infarction. A. Representative c-kit stained sections 3d after coronary artery ligation and saline or mini-TyrRS treatment. Arrows point to typical c-kit positive cells. B. Quantitation of c-kit positive cells/image; values shown are mean ± SEM (n = 3 mice/group; 15–16 images were quantified/mouse; *P<0.01). Bar is 100 um.

The discovery of proliferating myocytes in the human heart after infarction has led to the identification of resident cardiac progenitor cells [30]; multiple cell types have been identified as progenitors based on the expression of stem cell markers, including the tyrosine kinase receptor, c-kit [31], [32]. Because of the known chemoattractant cytokine activity of mini-TyrRS [3]–[5], [33], we tested the intriguing hypothesis that mini-TyrRS may act as a ‘homing’ signal to promote the recruitment of c-kit^pos^ cells to the infarcted heart. We examined the presence of c-kit^pos^, cells by immunostaining heart tissues with a c-kit antibody (Figure 6). Our results show that mini-TyrRS increases the abundance of c-kit^pos^ cells in the area surrounding the infarct, suggesting that mini-TyrRS may promote the recruitment of these cells to the infarcted heart to aid in recovery after ischemia. We also examined possible differences in leukocyte migration in mini-TyrRS- and saline- treated hearts after infarction by immunostaining for CD45, a pan-leukocyte marker (Figure S1). We found no significant differences in leukocyte accumulation in the infarct or peri-infarct areas of the heart after MI in the two treatment groups.

## Discussion

In this study, we present evidence that systemic delivery of the biologic mini-TyrRS has angiogenic and cell-protective effects that ultimately result in improved cardiac recovery in a murine myocardial infarction model. After acute myocardial infarction in mice, mini-TyrRS resulted in increased angiogenesis, decreased cell apoptosis, increased proliferation and recruitment of c-kit^pos^ cells, providing potential mechanisms for the improved cardiac function observed.

Previous reports have demonstrated the chemotactic effects of mini-TyrRS on polymorphonuclear leukocytes [33], as well as its pro-angiogenic effects on ECs [4], [5]. The angiogenic effects of mini-TyrRS have also been demonstrated *in vivo* [5], [34], [35] using the chicken chorioallantoic membrane (CAM) assay; mouse matrigel assay; and two models of ischemia. Our own laboratory has provided a mechanistic basis for the action of mini-TyrRS in angiogenesis: we have shown that mini-TyrRS activates three key regulatory signaling pathways in ECs: ERK, Akt and Src, and stimulates EC proliferation and migration [4]. Mini-TyrRS was also found to induce activation of eNOS and a transient increase in EC permeability [4]. Collectively, these studies demonstrated the proangiogenic properties of mini-TyrRS and allowed us to hypothesize that mini-TyrRS is beneficial in the clinically-relevant setting of myocardial infarction.

The cardiac improvement in response to mini-TyrRS treatment suggests that preservation of myocardial tissue and the concurrent increase in microvascular density may be involved in the observed cardiac recovery. The rapid improvement of cardiac function after mini-TyrRS treatment raises the possibility that mini-TyrRS preserves myocyte viability via cell-autonomous effects. Mini-TyrRS is a survival signal for ECs, but a similar activity on cardiomyocytes has not been demonstrated. Alternatively, it is also possible that mini-TyrRS treatment rescues cardiomyocytes at the ischemic border zone via vasodilation of residual vessels; indeed, we have previously shown that mini-TyrRS induces activation of the vasodilatory enzyme eNOS in ECs [4]. Enhanced NO bioavailability has also been linked to attenuation of vessel loss and stimulation of angiogenesis during myocardial infarction [36].

The presence of stem cells in the heart after infarction is now well-documented and has received much attention [31]. In particular, c-kit^pos^ cells have been identified in the adult mammalian heart after infarction [30], [32] and have also been shown to have therapeutic effects when injected into mouse, rat and porcine infarct models [31], [37], [38]. Although the mechanisms underlying the therapeutic effects of cardiac stem cells are still under investigation, as well as contention, it has been shown that following injection of cardiac stem cells, large numbers of newly formed, albeit immature, cardiomyocytes are seen [39]. Additionally, substantial numbers of arterioles and capillaries are also seen, suggesting that cardiac stem cells can also differentiate into ECs [31]. Although we do not know at this point the fate of the c-kit^pos^ cells recruited in response to mini-TyrRS treatment, we hypothesize that the presence of these cells in the remodeling hearts contributes to cardiac regeneration and recovery. It is also possible that mini-TyrRS stimulates recruitment and proliferation of cardiac fibroblasts that secrete factors that promote angiogenesis and cardiomyocyte survival. Therefore, cardiac improvement associated with mini-TyrRS treatment may be multi-factorial, involving effects on various resident cardiac cells and effects on tissue survival, angiogenesis and stem cell recruitment.

The effects of mini-TyrRS on stimulating fibroblast proliferation, yet reducing scar tissue compared to saline-treated hearts might on first look seem contradictory. One of the well-known functions of cardiac fibroblasts is the synthesis and breakdown of extracellular matrix components [28]; this process is particularly important during the cardiac remodeling that follows an infarction. Degradation of collagen and other extracellular matrix constituents requires activation of MMPs; interestingly, we have previously shown that mini-TyrRS activates MMP2 in vascular ECs [4]. In the early stages following cardiac injury, infiltrating inflammatory cells secrete cytokines and growth factors that promote fibroblast activation, proliferation and differentiation, followed by deposition of new matrix proteins and scar formation. At this stage, increases in matrix proteins and scar tissue is considered reparative, as it serves to replace areas of cardiomyocyte loss; however, if this process persists uncontrollably, it may become maladaptive and ultimately lead to reduced cardiac function [40]. Our data suggest that while mini-TyrRS promotes cardiac fibroblast proliferation and recruitment to the site of injury, this process is tightly regulated so that it results in reduced scar tissue and increased cardiac function. In fact, the cardiac fibroblast is not just a bystander that functions merely to increase fibrosis; it has recently been shown that cardiac fibroblasts are crucial players in cardiac adaptive responses [41], possibly via secretion of paracrine factors [42] that result in cardiomyocyte hypertrophy and overall cardiac protective effects [43], [44].

Our research explores the potential for the naturally occurring biologic mini-TyrRS to act as a therapeutic for the treatment of myocardial infarction. There are several properties of mini-TyrRS that make it an attractive candidate for therapeutic development. First, we administered mini-TyrRS by IP injection rather than intracardially at the site of injury. Secondly, in contrast to the early administration of most other cytokines or growth factors before coronary artery ligation, mini-TyrRS was administered a few hours after infarction, therefore more closely resembling a clinically-relevant setting. Finally, development of therapeutics based on naturally-occurring secretory biologics, such as mini-TyrRS, might provide a new strategy for developing novel therapeutics with minimal unwanted side-effects.

The last decade has seen skyrocketing interest in the field of tRNA synthetase biology, especially their expanding participation in multiple cellular functions and their increasing appreciation as causative agents for human diseases. Our study reveals that delivery of mini-TyrRS after infarction clearly provides therapeutic *in vivo* benefits and adds to the repertoire of non-canonical aaRS functions.

## Supporting Information

Figure S1 Effects of mini-TyrRS treatment on CD45-positive cells in the heart after infarction. A. Representative CD45 stained sections after coronary artery ligation and saline or mini-TyrRS treatment. Areas at the infarct and peri-infarct sites are shown. B. Quantitation of CD45 positive cells/image; values shown are mean ± SEM (n = 3/group; 10 images/mouse). Bar, 80 um.
(DOCX)
