# Supplementary material for: Natural Aminoacyl tRNA Synthetase Fragment Enhances Cardiac Function after Myocardial Infarction
Source: PLoS One. 2014 Oct 8;9(10):e109325. doi: 10.1371/journal.pone.0109325 (PMC4190278; doi:10.1371/journal.pone.0109325)
Supplement: Figure S1 — Effects of mini-TyrRS treatment on CD45-positive cells in the heart after infarction. A. Representative CD45 stained sections after coronary artery ligation and saline or mini-TyrRS treatment. Areas at the infarct and peri-infarct sites are shown. B. Quantitation of CD45 positive cells/image; values shown are mean ± SEM (n = 3/group; 10 images/mouse). Bar, 80 um. (DOCX) [file pone.0109325.s001.docx]

**Supplementary Figure 1. Effects of mini-TyrRS treatment on CD45-positive cells in the heart after infarction.** A. Representative CD45 stained sections after coronary artery ligation and saline or mini-TyrRS treatment. Areas at the infarct and peri-infarct sites are shown. B. Quantitation of CD45 positive cells/image; values shown are mean ± SEM (n=3/group; 10 images/mouse). Bar, 80um.
